# *GhMYB106_D13* coordinates cuticle biosynthesis to build the dual shields for anther protection in upland cotton

**DOI:** 10.1111/tpj.71142

**Published:** 2026-10-07

**Authors:** Bin Gao, Meilin Chen, Chao Fu, Xianlong Zhang, Zhongxu Lin

**Affiliations:** ^1^ National Key Laboratory of Crop Genetic Improvement College of Plant Sciences & Technology, Huazhong Agricultural University Wuhan Hubei 430070 China; ^2^ Guangdong Provincial Key Laboratory for Plant Epigenetics College of Life Sciences and Oceanography, Longhua Bioindustry and Innovation Research Institute, Shenzhen University Shenzhen Guangdong 518060 China; ^3^ Xinjiang Uygur Autonomous Region Academy of Agricultural Sciences Urumqi 830091 China

**Keywords:** cotton, MYB106, anther, cuticle, trichome

## Abstract

The plant cuticle acts as a crucial barrier against abiotic stresses. In this study, the *vulnerable anther* (*van*) gene on chromosome D13 was map‐based cloned in upland cotton and identified as *GhMYB106_D13*. It was demonstrated that the differential distribution of *MYB106* between D13 and A13 may represent post‐polyploidization convergent evolutionary events. Transcriptome analysis, metabolic profiling, and microscopic examination of anther cuticle in *GhMYB106_D13* over‐expression and knockout lines revealed that *GhMYB106_D13* affects the wax and cuticle biosynthesis pathway. GC‐MS analysis demonstrated that the loss of *GhMYB106_D13* resulted in an ~27.6% reduction in total wax load, predominantly characterized by a decrease in very‐long‐chain alkanes, whereas overexpression (OE) led to wax accumulation. Key genes involved in the pathway including *FACR2*, *LACS1*, *HHT1*, and *PXG4* were found to be changed dynamically. Specifically, *GhMYB106_D13* likely activates the expression of *LACS1*, a gene involved in wax synthesis that was downregulated in Stage‐8 anthers of the *Ghmyb106_D13* mutant. Meanwhile, upregulation of *FACR2*, *CYP704B1*, and *HHT1* was suggested to be potentially involved in cuticle metabolism, possibly through altered 16‐feruloyloxypalmitic acid (16‐FA) level. Thus, *GhMYB106_D13* contributes to the formation of the first protective ‘shield’ for the anther against external stresses. Additionally, *GhMYB106_D13* participates in trichome differentiation, which constitutes a second ‘shield’ that encloses the petals and sequesters the anther in the bud before flowering. Overall, these findings unveil the dual function of *GhMYB106_D13* in physical protection of plant propagation and offer valuable insights for future strategies aimed at improving stress tolerance in crops.

## Significance Statement

Understanding how plants defend their sensitive reproductive organs against environmental stresses is crucial for plant breeding. We demonstrate that the transcription factor *GhMYB106_D13* coordinates microscopic cuticular wax synthesis and macroscopic bud‐enclosing trichomes, offering key developmental insights and potential genetic targets for improving abiotic stress tolerance in plants.

## INTRODUCTION

In terrestrial plants, the transition from vegetative growth to reproductive development represents a critical life‐history phase that is exquisitely sensitive to environmental fluctuations. The male gametophyte, housed within the anther, is particularly vulnerable to abiotic stresses such as drought, heat, and ultraviolet (UV) radiation (Borg et al., 2009; Collin et al., 2008). To ensure reproductive success, plants have evolved sophisticated physical and chemical barriers to sequester developing anthers from external aggressions. The primary interface mediating this protection is the cuticle, a hydrophobic extracellular layer composed of a cutin polyester matrix embedded with and coated by cuticular waxes (Yeats & Rose, 2013). Defects in this ‘physiological shield’ often lead to uncontrolled non‐stomatal water loss, anther dehydration, and subsequent male sterility, as evidenced by mutants in rice (*Oryza sativa*) and maize (*Zea mays*) (Chen et al., 2017; Jung et al., 2006; Wu et al., 2014; Yang et al., 2014). While the biosynthetic pathways of cuticular components involving fatty acid elongation and modification in the endoplasmic reticulum are well conserved (Bernard & Joubès, 2013; Samuels et al., 2008), the precise transcriptional networks that coordinate these metabolic fluxes with epidermal cell differentiation during floral development still need further elucidation.

Transcriptional regulation of cuticle biosynthesis is dominated by the R2R3‐MYB superfamily. In *Arabidopsis thaliana*, specific MYB factors, such as *MYB96* and *MYB94*, activate wax biosynthesis to confer drought tolerance (Lee & Suh, 2015; Seo et al., 2011). Recent studies have highlighted the pivotal role of Subgroup 9 MYBs, particularly MIXTA‐like proteins, in this process. Oshima and Mitsuda (2013) demonstrated that *AtMYB106* (also known as *NOECK*) and its paralog *AtMYB16* coordinately regulate cuticle development in Arabidopsis and *Torenia fournieri* by acting upstream of *WAX INDUCER1/SHINE1* (*WIN1*/*SHN1*). This regulatory module appears to be environmentally responsive; for instance, Jiang et al. (2024) recently revealed that, in apple (*Malus domestica*), the BTB‐TAZ protein MdBT2 responds to high nitrogen supply by degrading *MdMYB106*, thereby downregulating the acyl‐transferase gene *MdCER2L1* and reducing wax accumulation. Beyond metabolic regulation, *MYB106* is also integrated into developmental timing, as Hong et al. (2021) showed that it functions as a negative regulator of flowering time in Arabidopsis by repressing *FLOWERING LOCUS T* (*FT*). These findings suggest that *MYB106* serves as a central hub linking environmental cues, developmental phase transitions, and cuticle biosynthesis.

A defining characteristic of MIXTA‐like transcription factors is their dual functions in regulating both cuticle biochemistry and epidermal cell morphology, particularly trichome initiation and branching. Folkers et al. (1997) identified the *noeck* (*myb106*) mutant in Arabidopsis, which displays over‐branched trichomes, establishing its role as a repressor of branching. Subsequent transcriptional profiling confirmed that *AtMYB106* is a key regulator of trichome maturation (Jakoby et al., 2008). This regulatory network involves complex protein–protein interactions; for example, the Class I TCP transcription factor *TCP15* has been shown to physically interact with *MYB106* to modulate trichome development and cuticle gene expression (Camoirano et al., 2021). In upland cotton (*Gossypium hirsutum*), an allotetraploid crop valued for its seed trichomes (fibers), MIXTA‐like genes have undergone functional diversification. While *GhMYB25‐like* (a MIXTA homolog) is critical for seed fiber initiation (Walford et al., 2011), other homologs regulate distinct trichome types. Tan et al. (2016) discovered that *GhMYBML10* (an ortholog of *MYB106*) regulates trichomes on the petal margins, which is entangled to form a ‘Velcro‐like’ mechanism of keeping the flower bud closed and maintaining internal structural integrity. Further evolution of these regulatory networks has led to the development of specialized trichomes at the petal base in *Malvoideae* species (Tan et al., 2020). Recently, the *Open‐bud* (*ob*) mutant in cotton, which displays exposed stigmas and anthers due to abnormal petal development, was mapped to the *MML10* locus, confirming the conservation of this gene family in controlling floral architecture (Chen et al., 2020).

Despite these advances, the specific contribution of *MYB106* orthologs to anther protection in polyploid crops remains underexplored. The ‘Velcro’ mechanism of petals provides a physical ‘second shield’ for the flower bud (Tan et al., 2016), but when this barrier fails (as in *ob* mutants) or when the flower opens, the ‘first shield’ of anther surface cuticle becomes the sole defense against desiccation. It is unclear whether *MYB106* coordinately regulates both the macroscopic physical enclosure (via petal/trichome development) and the microscopic chemical barrier (anther cuticle) to ensure male fertility. In this study, a cotton mutant, *vulnerable anther* (*van*), was identified which is characterized by necrotic anthers and sensitivity to environmental stress. Through map‐based cloning, *GhMYB106_D13* was identified as the causal gene located on chromosome D13. By integrating transcriptomic analysis with metabolic profiling of cuticular waxes, *GhMYB106_D13* was shown to activate the biosynthesis of very‐long‐chain alkanes. Our findings support a ‘dual‐shield’ model where *GhMYB106_D13* is essential for establishing both the anther cuticle (first shield) and the trichome‐mediated floral enclosure (second shield), thereby providing a multilayered defense strategy for plant propagation.

## RESULTS

### Identification of *vulnerable anther* mutant in cotton

The *vulnerable anther* (*van*) mutant, derived from M247, is an introgression line derived from a cross between E22 and 3‐79. Introgressed segments were identified on chromosome D13 (Table S1). Mutant plants exhibited improperly closed petals at early developmental stages. Under field conditions, anthers located in the upper part of the flower were exposed to sunlight approximately 1 week before blooming, became necrotic, and turned black (Figure 1a,b). An additional mutant, EH191, an introgression line in the E22 background from *Gossypium mustelinum* (AD_4_), displayed similar phenotypes (Figure 1c); however, no *van* mutant was identified in introgression lines of *Gossypium tomentosum* (AD_3_) (Keerio et al., 2018). Overlapping introgressed segments collocating with the M247 introgression region were confirmed in EH191 (Table S1).

**Figure 1:**
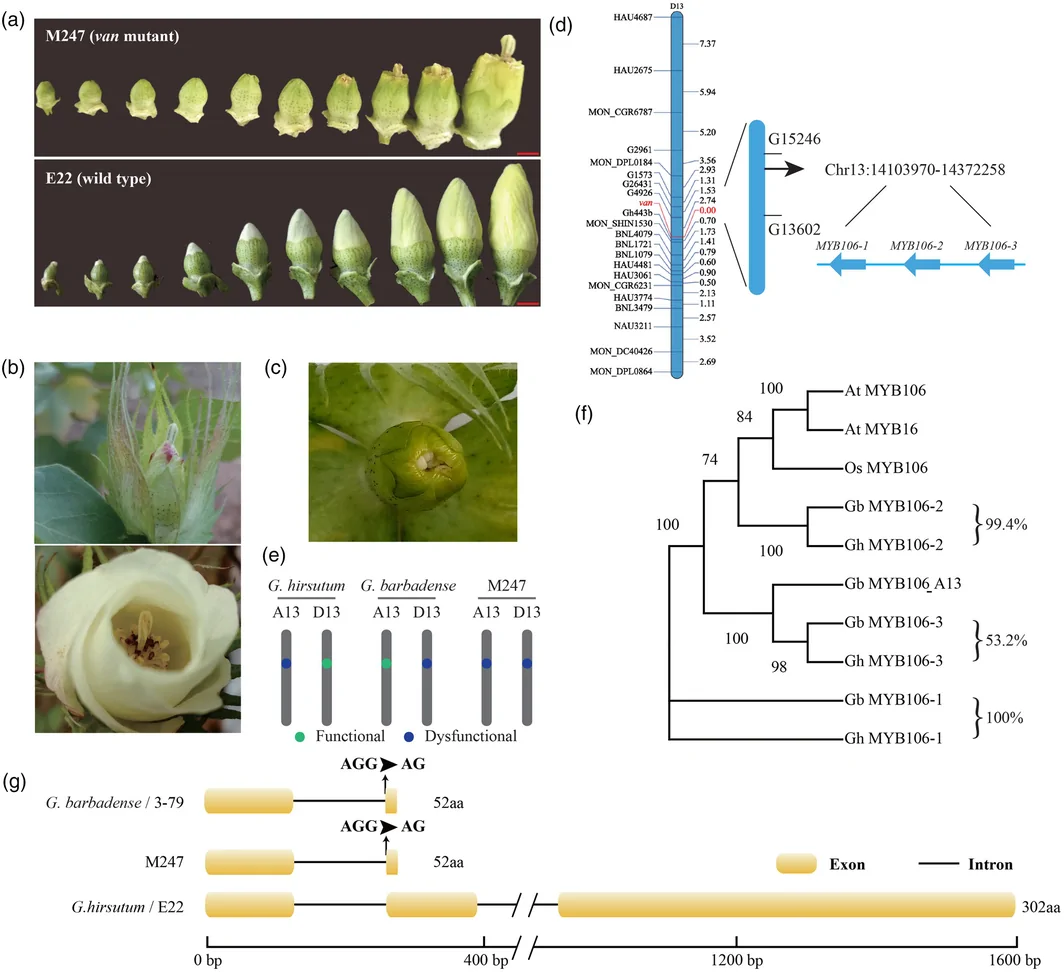
Phenotypic characterization and map‐based cloning of the *van* gene. (a) Comparison of flower bud development between the *van* mutant (top) and the wild type (bottom). Bar = 0.5 cm. (b, c) Flower buds of M247 (b) and EH191 (c). (d) Map‐based cloning of the *van* locus on Chromosome D13. (e) Distribution of *van* locus in *Gossypium hirsutum* (*Gh*), *Gossypium barbadense* (*Gb*), and M247. (f) Phylogenetic tree of MYB106_D13 proteins. The phylogenetic relationship of MYB106_D13 homeologs from *G. hirsutum* (*Gh*), *G. barbadense* (*Gb*), *Arabidopsis thaliana* (*At*), and *Oryza sativa* (*Os*) is shown. Numbers indicate the percentage of amino acid sequence identity between homologous pairs. (g) Gene structure and mutation analysis of *GhMYB106_D13*.

### Genetic mapping of *van*

A single recessive genetic locus was deduced to control the vulnerable anther phenotype, as the separation ratio of 396:118 (normal anther phenotype:vulnerable anther phenotype, ~3:1, *χ* ^2^ = 0.477, *P* < 0.05) in F_2_ population of M247 × E22. For mapping of the controlling gene, genome‐wide molecular markers showing polymorphism in an F_2_ population containing 1569 individuals were screened. A total of 22 polymorphic markers were found on chromosome D13, and the candidate gene was mapped to a 1.6‐Mb region (Figure 1d). This interval exactly encompasses the classical *ob1* locus, which was previously implicated in petal trichome initiation (Tan et al., 2016). Given that the *van* mutant shares strikingly similar floral phenotypes with the *ob1* mutant, specifically the defective petal closure and subsequently exposed anthers, it was strongly deduced that the *van* mutation is allelic to *ob1* or controlled by the same gene.

Indeed, it has been previously reported that the *ob1* trait is controlled by duplicate loci on homoeologous chromosomes A13 and D13, which exhibit reciprocal dominance in tetraploid cottons (Chen et al., 2020). Specifically, *Gossypium barbadense* (e.g., 3‐79) possesses a functional (dominant) allele on A13 but a non‐functional (recessive) allele on D13, whereas *G. hirsutum* (e.g., E22) displays the opposite pattern. Consequently, the *van* mutant M247, which is a *G. hirsutum* introgression line harboring a *G. barbadense* chromosomal segment on D13, carries recessive alleles at both loci. To validate the function of the A13 homeolog, an additional F_2_ population derived from a cross between M247 and 3‐79 was generated. As anticipated, the F_2_ population segregated for the *van* phenotype with a ratio of 198:61 (normal anther phenotype: vulnerable anther phenotype, ~3:1, *χ* ^2^ = 0.326, *P* < 0.05), consistent with the segregation of a single recessive gene on A13. Furthermore, several co‐segregating markers were identified within the collinear region on chromosome A13 (Table S1), confirming the presence of the functional *van* homeolog in the A13 subgenome of *G. barbadense* (Figure 1e). In summary, both of the loci on A13 and D13 are recessive in M247, which results in the *van* phenotype; while in *G. hirsutum* (AD_1_) and *G. barbadense* (AD_2_), one of the homoeologous loci is dominant, and the *van* phenotype disappears (Figure 1e).

Within the mapped region on D13, a total of 28 genes were actively expressed (Li et al., 2024). Candidate gene screening involved comparing these 28 genes between AD_1_ and the corresponding regions in AD_2_ and the collinear region on AD_1_‐A13 (Table S2). Four genes matched the expected pattern, with *MYB106‐3* locating in a three‐gene cluster and getting the best match (Table S2). The MYB106‐3 protein in AD_2_ is truncated compared with that in AD_1_ (Figure 1f,g). mRNA sequencing revealed a consistent G‐to‐A mutation at the 3′ splice site of the first intron in *van* mutant and 3‐79, resulting in a 1‐bp deletion in the second exon and a truncated coding sequence (CDS) (Figure 1g; Figure S1A). This mutation of *MYB106‐3* was consistent with that of *ob1* mutant, which confirmed that the *van* mutant harbors a loss‐of‐function mutation in *MYB106‐3* (*GhMML10*), demonstrating that *van* is allelic to *ob1*.

Conclusively, the *MYB106‐3* gene was the ideal candidate. It is worth mentioning that the *MYB106‐3* gene is stable in diploid genomes (Figure S1B); in *G. mustelinum* (AD_4_), the key mutation in *MYB106_D13* is a 5354‐bp insertion in the second exon resulting in the disruption of this gene, whereas the homoeologous copy of A13 remains intact. Additionally, transcriptome analysis of *G. mustelinum* (accession number SRR1174699) revealed expression of only the A13 homeolog. *Gossypium darwinii* (AD_5_) and *Gossypium stephensii* (AD_7_) carry the same key mutation as *G. barbadense* (a 1‐bp deletion in the second exon) (Peng et al., 2022; Wang, Wendel, & Hua, 2018; Yang et al., 2023). *Gossypium ekmanianum* (AD_6_) possesses an intact D13 copy but lacks the A13 homeolog (Peng et al., 2022), as in *G. hirsutum*. In contrast, *G. tomentosum* (AD_3_) possesses intact MYB106 genes in both the A13 and D13 subgenomes (Shen et al., 2021) (Figure S1C). Reasonably, the localization of the *van*‐controlling *MYB106* copy on A13 or D13 in AD_1_, AD_2_, AD_4_, AD_5_, AD_6_, and AD_7_ was likely due to the post‐polyploidization event and/or convergent evolution.

### Functional validation of *GhMYB106_D13*

*MYB106‐3* (hereinafter referred to as *GhMYB106_D13*) showed developmentally dynamic expression across examined tissues, with notable enrichment in ovules (Figure 2a). The expression level of *GhMYB106_D13* was higher in Stage‐8 anthers (tetrad stage) than in mature anthers, a trend also observed in fibers at 5 and 10 days post‐anthesis. Subcellular localization confirmed that GhMYB106_D13 is nuclear‐localized, supporting its role as a transcription factor (Figure 2b).

**Figure 2:**
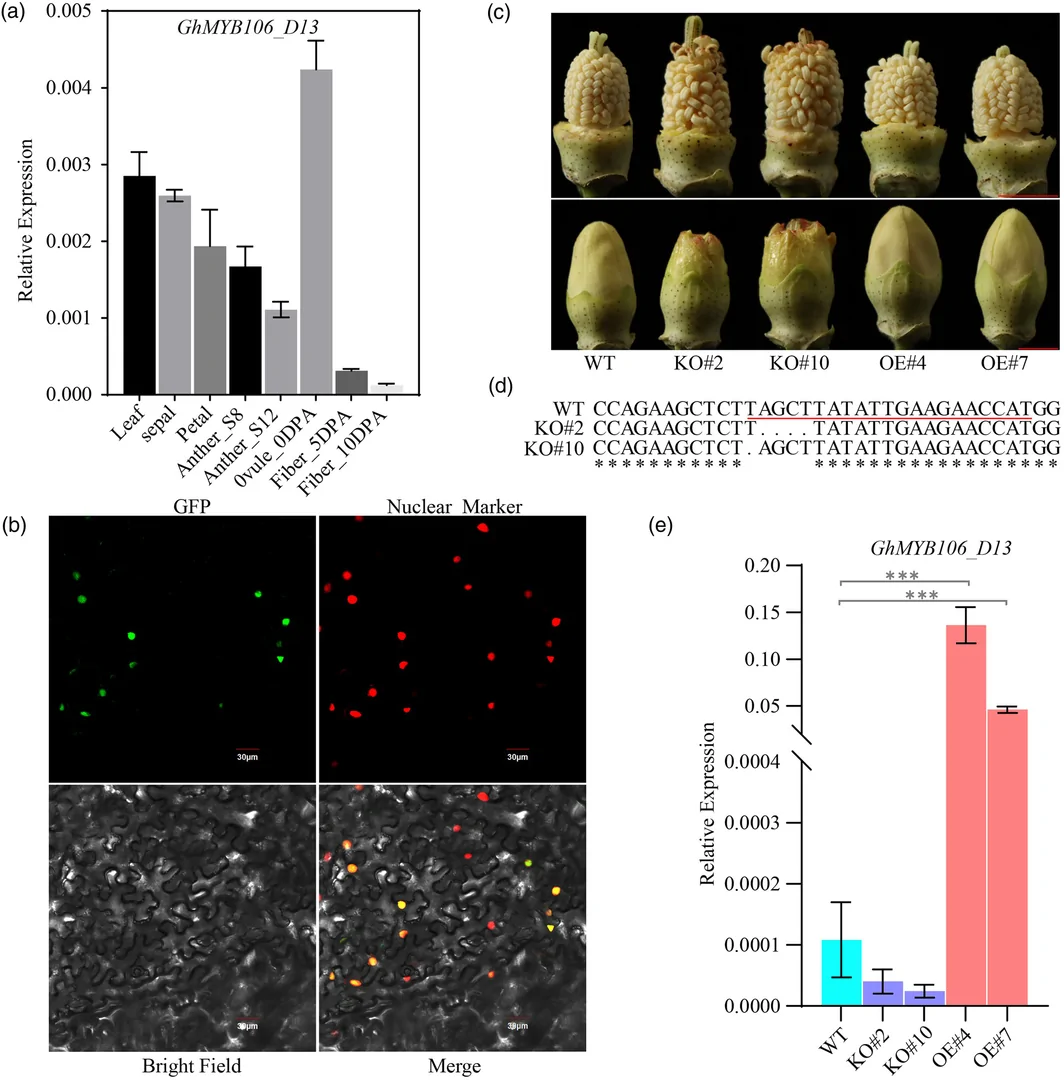
Expression profile, subcellular localization, and functional validation of *GhMYB106_D13*. (a) Tissue‐specific expression pattern of *GhMYB106_D13* in *Gossypium hirsutum* acc. Jin668. Data represent the mean ± SD of three biological replicates. (b) Subcellular localization of GhMYB106_D13. 4′,6‐Diamidino‐2‐phenylindole (DAPI) was used as the nuclear marker (red). (c) Phenotypic characterization of *GhMYB106_D13* transgenic lines. The top row shows anthers with petals removed, and the bottom row shows intact flower buds just before anthesis. Bar = 1 cm. (d) Genotyping of CRISPR/Cas9‐induced mutations. The sgRNA target sequence is indicated by the red underline. (e) Verification of *GhMYB106_D13* transcript levels in wild‐type (WT), knockout (KO), and overexpression (OE) transgenic lines by qRT‐PCR. Asterisks indicate significant differences (***P < 0.001, Student's *t*‐test).

To understand the role of *GhMYB106_D13*, two knockout (KO) lines and two OE lines were developed with genetic background of an upland cotton accession Jin668. As expected, the KO lines displayed phenotypes resembling those of *van* mutants under field conditions: the loosely closed petals and vulnerable up‐part anthers (Figure 2c). No other obvious different phenotypes of KO lines were found before flowering (Figure S2). Sequencing confirmed a 4‐bp deletion and 1‐bp deletion in the first exon of *MYB106_D13* in KO#2 and KO#10, resulting in truncated proteins of 51 and 23 amino acids, respectively (Figure 2d). *GhMYB106_D13* expression level was significantly upregulated in the −1 DPA anther of OE lines (Figure 2e), which had normal buds and anthers (Figure 2c).

### Wax and cuticle biosynthesis pathway is shaped by *GhMYB106_D13*

For investigating the molecular basis of *van* phenotypes and capturing the dynamic transcriptional changes during the development of *van* phenotypes, transcriptomes of Stage‐8 and Stage‐12 anthers from KO and OE lines were compared with wild‐type (WT) Jin668. In Stage‐8 anthers, a total of 2009 and 1670 differentially expressed genes (DEGs) were identified in KO and OE, respectively (|fold change| ≥2) (Figure 3a,b). Among the 602 common DEGs, Gene Ontology (GO) enrichment analysis highlighted terms related to phenylpropanoid metabolism, fatty acid metabolism, and suberin biosynthetic processes (Figure 3c). Specifically, KEGG analysis revealed significant enrichment of the ‘Cutin, suberine, and wax biosynthesis’ pathway, followed by ‘Phenylpropanoid biosynthesis’ (Figure 3d). Additionally, in KO lines, enriched genes in the ‘Cutin, suberine, and wax biosynthesis pathway’ were mostly downregulated (Figure 3e). Given that phenylpropanoids and very‐long‐chain fatty acid (VLCFA) derivatives are major components of cuticular waxes, and cutin is primarily composed of long‐chain fatty acid derivatives (C16–C18), the mechanisms of *van* phenotype caused by the mutation in *GhMYB106_D13* were pointed to the wax and cutin biosynthesis pathway.

**Figure 3:**
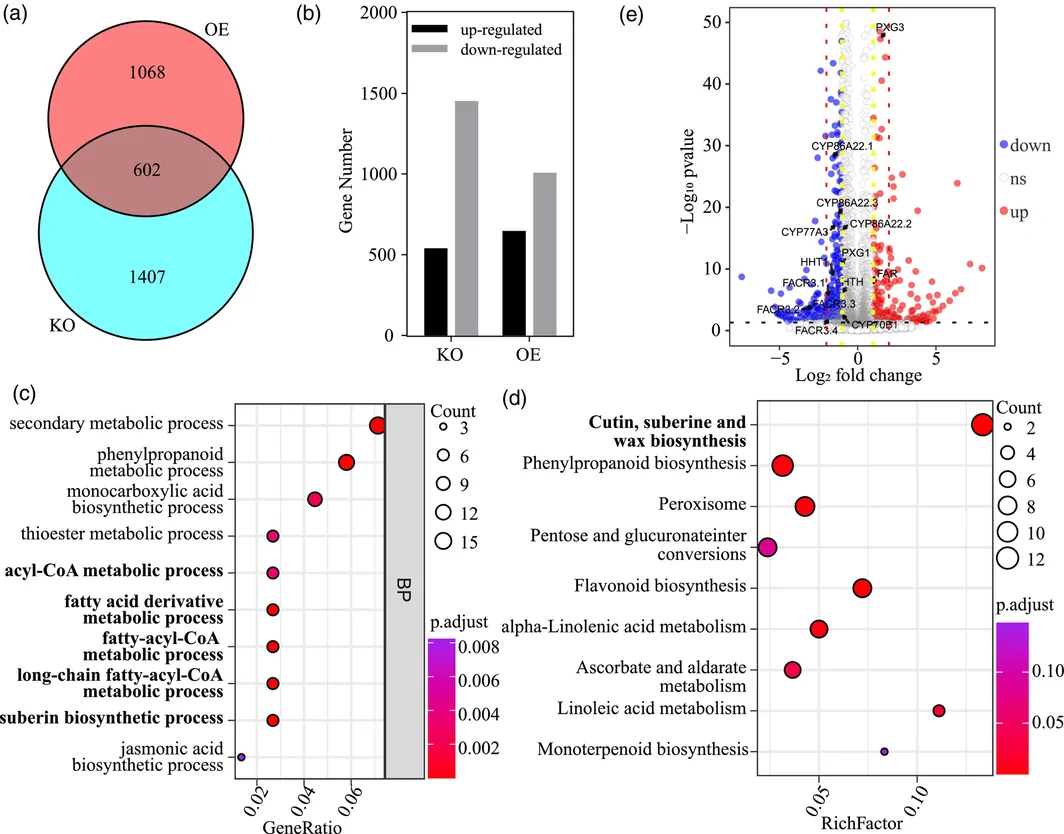
Transcriptomic analysis reveals that *GhMYB106_D13* regulates the wax and cutin biosynthesis pathway in cotton anthers. (a) Venn diagram shows the number of unique and overlapping differentially expressed genes (DEGs) in *GhMYB106_D13* knockout (KO) and overexpression (OE) lines compared with the wild type (WT) at Stage‐8 anther. (b) The number of upregulated (black) and downregulated (gray) DEGs in KO and OE lines. (c) Gene Ontology (GO) enrichment analysis of the 602 common DEGs shared by KO and OE lines. (d) KEGG pathway enrichment analysis of the common DEGs. (e) Volcano plot of DEGs identified in the *GhMYB106_D13* KO line. Red and blue dots indicate significantly upregulated and downregulated genes, respectively (|log2FC| >1, *P*‐adj <0.05). Key genes involved in the cuticle biosynthesis pathway are labeled.

In Stage‐12 anthers, the ‘Cutin, suberine, and wax biosynthesis pathway’ was also significantly enriched in KO and OE lines (Figure S3A), involving genes such as *HHT1*, *CYP704B1*, *CYP86A22*, *CER1*, *FACR2*, and *PXG4*. GO terms like pollen wall assembly or pollen exine formation were specifically enriched in KO and OE lines, with most associated genes upregulated in both KO and OE lines (Figure S3B). It was concluded that the consistent transcriptional change was a response to the vibration of wax and cutin accumulation.

Totally, 40 genes involved in wax and cutin biosynthesis were picked out from DEGs in both developmental stages, including the previously reported relevant genes *LACS1/2/6* (Zhao et al., 2019) and *IPE1* (Chen et al., 2017). Some DEGs (e.g., *FACR2*, *HHT1*, *IPE1*) were specifically expressed in Stage‐8 anthers, while others (e.g., *CYP77A3*, *CYP86A22*, *PXG1*, *PXG3*) showed higher expression in Stage‐12 anthers (Figure 4a,b). *FACR2*, *CYP704B1*, and *HHT1* were upregulated at the transcript level, which is consistent with a possible role in 16‐FA (cutin monomer) metabolism. 16‐FA accumulation has been proposed to negatively regulate cuticle biosynthesis (Shi et al., 2021). However, because this study only extracted soluble cuticular waxes and did not directly measure cutin monomers, and the effect on 16‐FA levels and their potential feedback on cuticle biosynthesis remains speculative. In addition, downregulation of *LACS1* and *CER1* likely reduced the VLCFA synthesis, as VLCFAs are key wax components. The expression levels of *LACS1*, *PXG4*, *FACR2*, and *HHT1* were checked by qRT‐PCR (Figure 4c). The dynamic expression of those genes implied the complex regulation network of wax and cutin biosynthesis.

**Figure 4:**
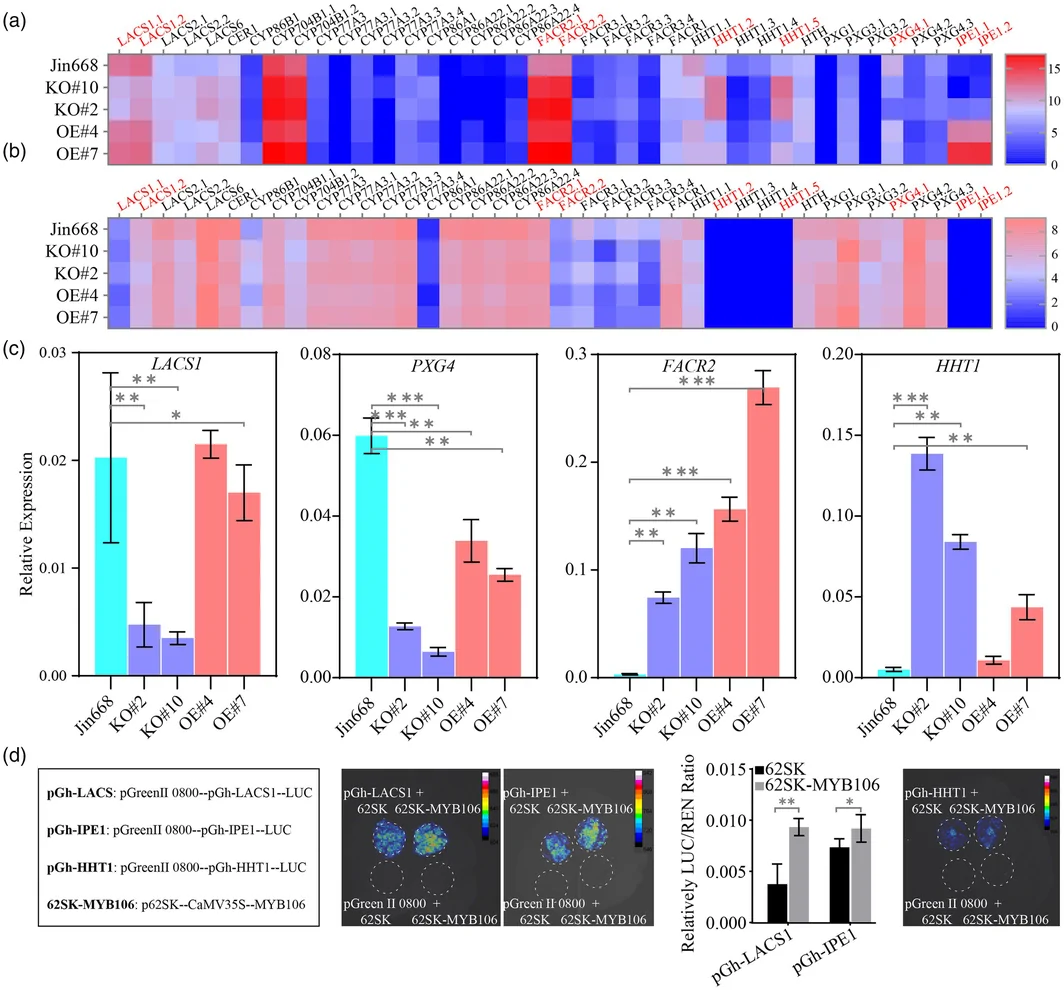
Expression profiles and transcriptional regulation of genes involved in wax and cutin biosynthesis. (a, b) Heat maps show the expression patterns of 40 selected genes related to wax and cutin biosynthesis in Stage‐8 (a) and Stage‐12 (b) anthers of wild type (Jin668), knockout (KO#2, KO#10), and overexpression (OE#4, OE#7) lines. (c) Quantitative RT‐PCR validation of the expression of *LACS1*, *PXG4*, *FACR2*, and *HHT1* in Stage‐8 anthers. Asterisks indicate significant differences (Student's *t*‐test). (d) Dual‐luciferase (Dual‐LUC) reporter assays show the transcriptional activation activity of *GhMYB106_D13*. The promoters of *GhLACS*, *GhIPE1*, and *GhHHT1* were fused to the LUC reporter, and *GhMYB106_D13* was used as the effector. Asterisks represent significant differences (Student's *t*‐test, *P < 0.05, **P < 0.01 and ***P < 0.001).

To determine whether *GhMYB106_D13* acts as a direct transcriptional activator within this complex network, *in vivo* dual‐luciferase reporter assays were performed. In this assay, GhMYB106_D13 was found to activate the expression of *LACS1* and *IPE1*, but not *HHT1* (Figure 4d). *LACS1* was highly expressed in WT Stage‐8 anthers, whereas *IPE1* expression remained low in both Stage‐8 and Stage‐12 anthers, similar to *GhMYB106_D13*. *IPE1* and *GhMYB106_D13* might be expressed in only a specific layer of cells near the anther surface. Promoter analysis by PlantCARE (https://bioinformatics.psb.ugent.be/webtools/plantcare/html/) revealed the presence of three MYB binding sites (sequence ‘YAACWG’) in the *LACS1* promoter and their absence in the *IPE1* promoter (Table S3), which suggests that GhMYB106_D13 may exert a direct activating effect on *LACS1* and indirect activation on *IPE1* through other effectors.

To definitively validate the metabolic impact of *GhMYB106_D13* on the anther cuticle, the cuticular wax load and composition in Stage‐12 anthers were quantified using GC‐MS (at this stage, the cuticular waxes are fully accumulated, and significant differences begin to appear in the mutant anthers). Consistent with the transcriptomic data, the chemical analysis revealed significant alterations in wax accumulation. The total wax load in KO lines decreased significantly to an average of 0.31 μg mm^−2^, representing an ~27.6% reduction compared with the WT (0.42 μg mm^−2^) (Figure 5a). Conversely, OE lines exhibited a significantly greater accumulation of wax, reaching an average of 0.71 μg mm^−2^ (Figure 5a). Compositional profiling identified alkanes, amides, and esters as the predominant wax classes (Figure 5b). Notably, alkanes, which constitute the primary barrier against water loss, were substantially reduced in KO anthers but dramatically elevated in OE lines (Figure 5b). Detailed chain‐length distribution analysis further showed that the abundance of dominant very‐long‐chain compounds, such as tetracosane (C24) and pentacosane (C25) (Figure 5c), was positively correlated with *GhMYB106_D13* expression levels. These metabolic profiles strongly support the hypothesis that *GhMYB106_D13* functions as a positive regulator of anther cuticular wax biosynthesis, likely by modulating key biosynthetic genes such as *LACS1* and *CER1*.

**Figure 5:**
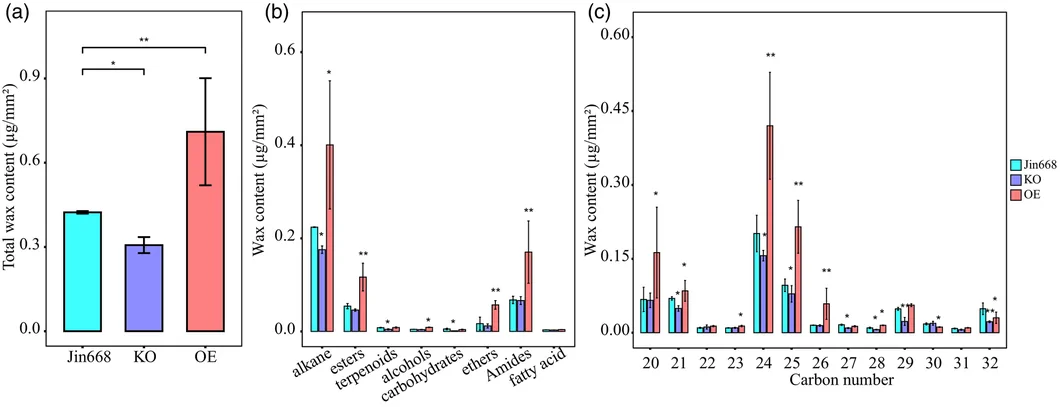
Analysis of cuticular wax content and composition. (a) Total cuticular wax of three genotypes. (b) The content of wax components classified by chemical class, including alkanes, esters, terpenoids, alcohols, ethers, carbohydrates, amides and fatty acids. (c) Distribution of wax constituents arranged by carbon chain length ranging from C20 to C32. Data are presented as means ± SD (*n* = 3 biological replicates). Asterisks indicate significant differences compared with Jin668 determined by Student's *t*‐test.

### *GhMYB106_D13* affects cuticle formation in anther

To determine whether Stage‐12 anthers have undergone morphological changes, scanning electron microscopy (SEM) was performed. As expected, anther cuticle morphology was significantly altered in OE and KO lines (Figure 6a). WT anthers displayed ridge‐like structures with clear, thread‐like cuticular patterning. In contrast, KO lines exhibited reduced, discontinuous, and faint cuticular threads, while OE lines showed densely packed thread‐like structures, indicating increased cuticle deposition. The pollen cuticle was also checked, but there was no obvious difference in KO or OE lines. Thus, *GhMYB106_D13* might have had a dosage‐dependent effect in cuticle accumulation of anther.

**Figure 6:**
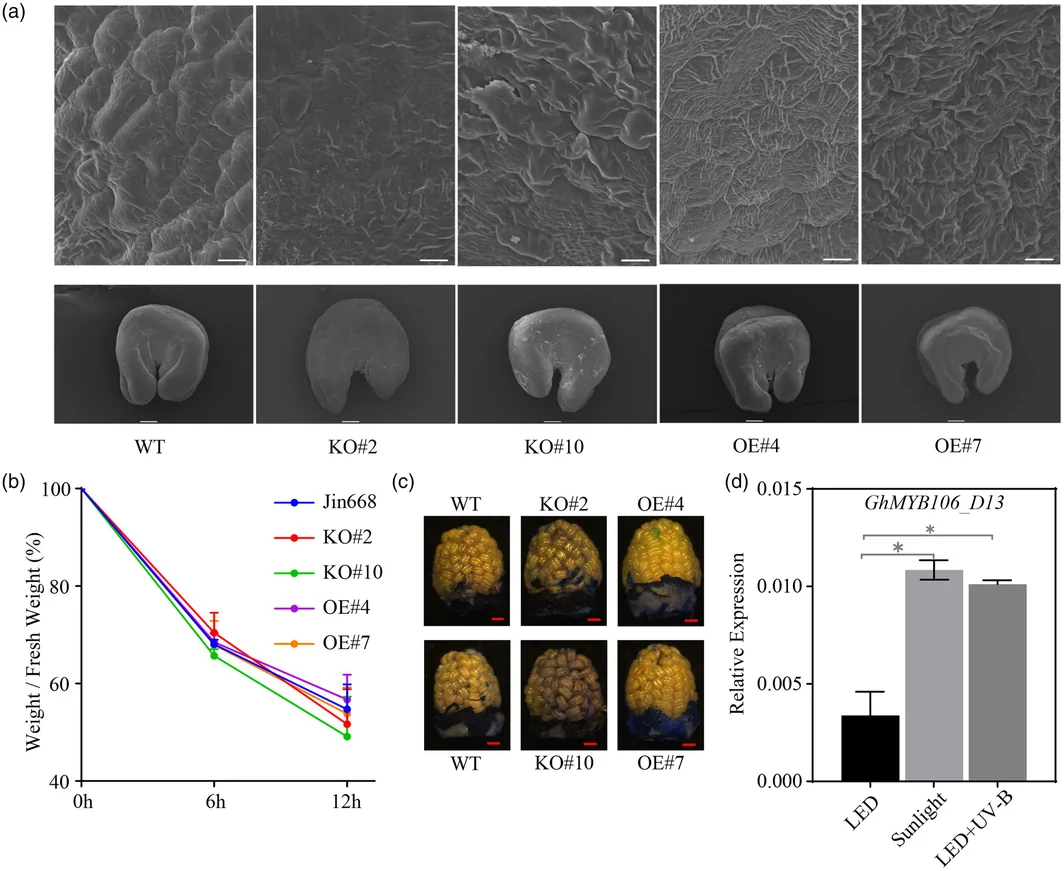
*GhMYB106_D13* regulates anther cuticle integrity. (a) Scanning electron microscopy (SEM) analysis of anther cuticle morphology at Stage‐12. The top row shows high‐magnification views of the anther epidermal surface (Bar = 10 μm). The bottom row shows low‐magnification views of whole anthers (Bar = 200 μm). (b) Water loss assay of detached anthers. (c) Anther cuticle permeability test. (d) Induction of *GhMYB106_D13* expression by environmental stimuli. Relative expression levels of *GhMYB106_D13* were measured in plants grown under LED light, natural sunlight, or LED supplemented with UV‐B light (Student's *t*‐test, *P < 0.05).

The cuticle serves as a protective shield against environmental stress, limiting non‐stomatal water loss and altering water permeability. The water loss rate in KO (average 49.58%) was found to be just slightly higher than in WT (average 45.25%), but no significant difference was observed (12 h, *P* = 0.58), and almost no difference found between OE (average 44.73%) and WT (12 h, *P* = 0.80) (Figure 6b). The rain‐simulating soaking experiment showed that anthers of KO lines were more permeable after 2 days of water immersion (Figure 6c).

The wax and cutin biosynthesis pathway could also interact with the environment. Formally, the *GhMYB106_D13* expression level was found to be elevated after 2 days when the plants were transferred from LED growth chamber to outdoor with sunlight; a similar upregulation was observed after LED+UV‐B treatment for 1 day (Figure 6d). In conclusion, *GhMYB106_D13* could respond to sunlight/UV‐B and positively regulate wax and cutin biosynthesis to alleviate environmental damage like immersion and UV‐B.

### *GhMYB106_D13* is involved in trichome differentiation

*GhMYB106_D13* was reported to regulate petal trichome differentiation in cotton (Tan et al., 2016). Consistently, reduced trichome density was observed on leaves and petals of KO lines. Conversely, OE lines exhibited enhanced trichome differentiation on leaves, petals, and even styles and seed (fuzz fibers), and the lint fibers showed no obvious difference among WT, KO, and OE (Figure S4). For anthers, the trichomes were the second ‘shield’ closing the petal and sequestering the anther to bud before flowering.

Transcriptomic analysis of the *ob* mutant (a *myb106* mutant in cotton) revealed enrichment of DEGs associated with both trichome differentiation and wax and cutin pathway (Chen et al., 2020). For trichome differentiation, 13 DEGs mainly functioned in cellulose synthesis, including *IRX*s, *DUF597*, and *GUX1*, were all downregulated in buds of the *ob* mutant (Table S4). For wax and cutin pathway, DEGs included *CYPs*, *FACRs*, *HHT1*, and *LACS1*, which were consistent with findings from anther transcriptomes of KO lines in our study (Figure S5A,B).

In summary, *GhMYB106_D13* acts as a key regulator in promoting cuticle biosynthesis in the anther, thereby constructing the primary protective shield for the anther. Similarly, in petals, *GhMYB106_D13* is also involved in the synthesis of the cuticle. These findings raise the possibility that, in addition to directly promoting trichome differentiation, *GhMYB106_D13* may indirectly facilitate and safeguard trichome cell differentiation through its role in enhancing cuticle formation, thereby establishing a secondary shield that ‘locks’ the petal via trichomes (Figure 7).

**Figure 7:**
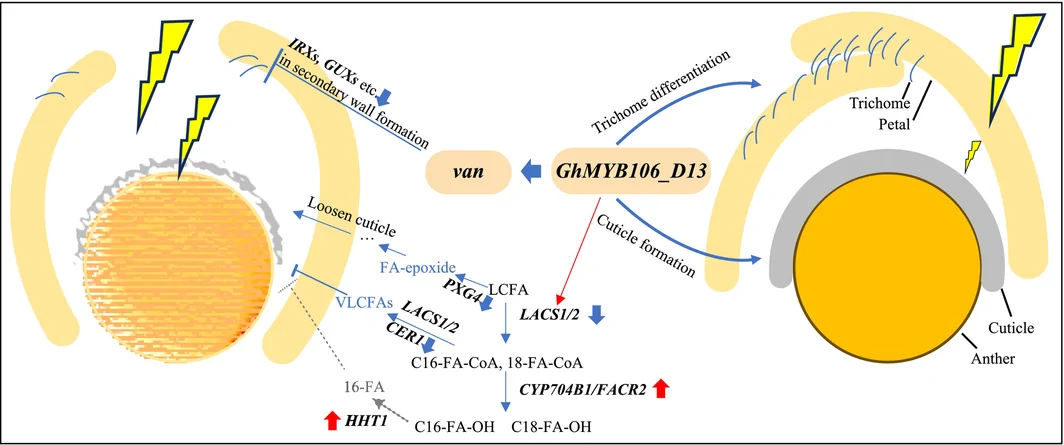
Proposed ‘Dual‐Shield’ model for *GhMYB106_D13*‐mediated anther protection. *GhMYB106_D13* coordinates a multilayered defense system: (1) It acts as a master regulator of the ‘First Shield’ (anther cuticle) by activating wax biosynthetic genes (e.g., *LACS1*) and possibly modulating lipid flux to prevent the accumulation of negative regulators (e.g., 16‐FA) (Shi et al., 2021); (2) it promotes the differentiation of petal trichomes, forming a ‘Second Shield’ that physically encloses the flower bud. In *van* mutant (left), the loss of *GhMYB106_D13* leads to a defective cuticle (looser structure) and reduced trichomes, rendering anthers vulnerable to environmental stresses like UV and desiccation. The red or blue lines/arrows indicate detected differences, and the gray lines/arrows indicate the predictions.

## DISCUSSION

### Metabolic reprogramming and non‐canonical lipid flux

Our combined transcriptomic and metabolomic analyses revealed that *GhMYB106_D13* acts as a crucial node in the metabolic network governing anther cuticle formation. The wax metabolomic analysis provides strong biochemical support for the transcriptomic evidence that *GhMYB106_D13* modulates biosynthesis through a dynamic reprogramming of lipid metabolism. In *GhMYB106_D13* OE lines, elevated total wax loads were accompanied by increased accumulation of amide‐type lipid compounds (Figure 5c). Although they are not canonical constituents of plant cuticular waxes, their enrichment likely reflects a highly active biosynthetic state where fatty acid flux exceeds the capacity of conventional esterification. Under such conditions, the wax precursor 16‐FA, promoted by the upregulation of *FACR2*, *CYP704B1*, and *HHT1* (Shi et al., 2021), may be redirected, reflecting the active state of lipid metabolism in these lines (Li‐Beisson et al., 2009). Conversely, *GhMYB106_D13* KO lines exhibited reduced wax accumulation, consistent with the downregulation of *LACS1* and *CER1* and diminished VLCFA synthesis (Bernard & Joubès, 2013; Lü et al., 2009). These findings suggest that *GhMYB106_D13* fine‐tunes lipid flux distribution, ensuring efficient cuticle formation.

### Biophysical implications: chain length and UV protection

The alteration in wax composition observed in our study has profound biophysical implications. As emphasized in a recent review by Heredia et al. (2024), the plant cuticle is not merely a passive barrier but a dynamic biophysical interface where chemical heterogeneity dictates function. Our data showed a significant reduction in long‐chain alkanes (C29) in *Ghmyb106_D13* mutants. According to Huang et al. (2017), cuticular water permeability is more strongly correlated with the weighted average chain length (ACL) of acyclic wax components than with the total wax load itself. Longer hydrocarbon chains increase the crystallinity and hydrophobicity of the wax barrier, thereby effectively restricting water loss. This view is further supported by recent findings in *Populus*, where qualitative shifts in wax composition, specifically the chain length of wax esters and alkanes, were identified as the primary determinants of cuticular transpiration (Grünhofer et al., 2024).

Therefore, the vulnerability of *van* mutant anthers to dehydration is likely driven by the specific loss of VLCFAs and alkanes, which compromises the integrity of the ‘physiological shield’ (Yeats & Rose, 2013). Furthermore, the necrotic blackening of *van* anthers under sunlight suggests a failure in photoprotection. The cuticular matrix, particularly when embedded with cyclic compounds and phenolics, acts as an effective screen against UV radiation (Heredia et al., 2024). The disrupted wax crystalline structure in the mutant likely increases UV transmittance, exposing the underlying reproductive tissues to lethal photo‐oxidative stress (Pfündel et al., 2008).

### Evolutionary divergence of MIXTA‐like genes in *Gossypium*

The observation that diploid cottons possess functional *MYB106* alleles, whereas *G. hirsutum* and *G. barbadense* exhibit reciprocal retention of functionality, strongly suggests that these loss‐of‐function mutations occurred independently after polyploidization and subsequent speciation. Whole‐genome duplication confers genetic redundancy, which relaxes purifying selection on individual gene copies, allowing one homeolog to accumulate deleterious mutations while the other maintains ancestral function, a process known as non‐functionalization or subgenome fractionation (Adams & Wendel, 2005; Wendel, 2000). Our findings align with recent pan‐genomic insights revealing that asymmetric subgenome evolution and divergent gene retention are pervasive drivers of genetic diversity in allotetraploid cottons (Li et al., 2024).

In the case of cotton *MYB106*, the alternative preservation of either the A13 or D13 homeolog implies that a single functional copy provides sufficient dosage for normal trichome and cuticle development. Furthermore, this ‘reciprocal silencing’ highlights the complexity of subgenomic coordination; as noted by You et al. (2023), such cryptic genetic variations remain masked in single species but can be unveiled through interspecific hybridization, offering both challenges and opportunities for precision breeding by manipulating subgenome‐specific favorable alleles. The *GhMYB106*‐controlled protective system, comprising the anther cuticle and petal trichomes, is indispensable for male fertility, as evidenced by the necrotic anther in the *van* mutant (no functional copy). Generally, an overdosage of *GhMYB106* (keeping both functional copies) would cause excessive wax loading and overly dense trichomes, which could decelerate anther dehydration/dehiscence, affect flower opening (these phenotypes are also found in OE‐*GhMYB106_D13* plants). It is also reported that hyperactivation of wax biosynthesis imposes a severe metabolic penalty or energy waste (Camoirano et al., 2020; Seo et al., 2011).

However, an exception to this ‘single‐copy’ economical balance is observed in *G. tomentosum* (AD_3_), a wild allotetraploid species endemic to the Hawaiian Islands (Shen et al., 2021). Genomic analysis reveals that *G. tomentosum* retains functional MYB106 copies on both the A13 and D13 subgenomes (Figure S1C), which precisely explains why the *van* mutant phenotype was completely absent in our AD_3_ introgression populations (Keerio et al., 2018). Morphologically, *G. tomentosum* is renowned for its exceptionally dense, silvery epidermal trichomes and robust heat tolerance (Shen et al., 2021). The intense UV radiation and harsh coastal conditions of its native Hawaiian habitat exerted a strong adaptive selection pressure favoring this ‘double dosage’. In such an extreme condition, the enhanced physical shield (dense trichomes) and chemical barrier (increased cuticular wax deposition) driven by retaining both functional *MYB106* copies are crucial for reflecting excessive UV light and preventing lethal desiccation. This robust defense system provided a critical survival advantage that outweighed the associated metabolic penalties.

Thus, while keeping just one functional copy after polyploidization achieves the right balance for most cotton species, it protects the anther effectively without the harmful effects of overproduction; the dual retention in *G. tomentosum* elegantly demonstrates how specific environmental constraints can dynamically shape subgenomic dosage evolution to maximize the reproductive fitness of the plant.

The functional characterization of *GhMYB106_D13* highlights a significant evolutionary divergence within the MIXTA‐like gene family. In Arabidopsis, *AtMYB106* (*NOK*) functions primarily as a regulator of trichome branching (Camoirano et al., 2020; Folkers et al., 1997; Jakoby et al., 2008). In contrast, our data indicate that, in cotton, *GhMYB106_D13* positively regulates the initiation number of trichomes on petals and leaves, rather than their branching morphology. This suggests that, while the recruitment of MIXTA‐like genes to epidermal differentiation pathways is conserved, their specific regulatory logic has diverged between *Brassicaceae* and *Malvaceae* (Machado et al., 2009).

A genome‐wide analysis of the MIXTA family in *Gossypium* identified *GhMML10* as the ortholog of *AtMYB106* (Wu et al., 2018). However, in the phylogenetic analysis presented by Wu et al. (2018), *MML10* appeared as a distinct clade and did not cluster tightly with *AtMYB106*, unlike other homologs. This discrepancy might be attributed to algorithmic artifacts arising from the rapid expansion and sub‐functionalization of the MIXTA family in allotetraploid cotton, or it may reflect a high degree of sequence divergence associated with the acquisition of novel functions (Zhang et al., 2015). Our study confirms that *GhMYB106_D13* (synonymous with *GhMML10*) is indeed the functional homolog responsible for the open‐bud phenotype mapped by Chen et al. (2020), establishing its conserved role in floral architecture despite sequence divergence.

### Regulation of fuzz fibers

Interestingly, OE of *GhMYB106_D13* resulted in a noticeable increase in fuzz fibers (Figure S4). Previous studies have identified *GhMML3* and *GhMML4* as primary regulators of fuzz and lint fibers initiation, respectively (Machado et al., 2009; Wu et al., 2018), but the expression of those genes showed no significant change in KO and OE lines according to transcriptomic analysis. The ability of *GhMYB106_D13* to promote fuzz development when overexpressed suggests a degree of functional redundancy or a potential feed‐forward regulatory capacity conserved among cotton *MIXTA‐like* paralogs. However, despite its slight basal expression in ovules, *GhMYB106_D13* does not appear to act as a primary regulator of natural seed fiber differentiation. Crucially, the *GhMYB106_D13* KO lines displayed no obvious defects in either lint or fuzz fibers (Figure S4C,D). Therefore, it is postulated that the hyper‐accumulation of fuzz fibers in the OE lines is a manifestation of ectopic activation. As a *MIXTA‐like* transcription factor with an intrinsic capacity to drive epidermal cell outgrowth, the artificially overexpressed *GhMYB106_D13* likely hijacked and hyper‐activated certain trichome development pathways in the ovular epidermis that are otherwise tightly suppressed during normal development. This underscores the complexity of the transcriptional network governing cotton fiber development, where multiple MIXTA factors cooperate to fine‐tune trichome density and type (Walford et al., 2011).

### Conclusion: the dual‐shield model

The coordinated formation of the ‘dual barrier’ is fundamentally rooted in the pleiotropic role of *GhMYB106_D13* as a master regulator of epidermal cell fate. In land plants, it has been reported that cuticular wax deposition and trichome morphogenesis are actually tightly coupled facets of epidermal differentiation through specific *MYB* genes (Lu et al., 2022; Oshima & Mitsuda, 2013; Shi et al., 2018). Our study extends this paradigm to the protection of reproductive organs in polyploid cotton. Crucially, our transcriptomic profiling provides direct molecular evidence for this coordinated regulation: the perturbation of *GhMYB106_D13* simultaneously reprogrammed distinct downstream networks. In the *Ghmyb106_D13* plants, the downregulation of genes was observed to be responsible for lipid and cutin biosynthesis (e.g., *LACS1*, *FACR2*, and *HHT1*) and those essential for trichome outgrowth and secondary cell wall thickening (e.g., cellulose synthesis‐related genes *IRXs* and *GUX1*). This dual transcriptional rewiring likely underlies the bidirectional genetic phenotypes observed in our KO and OE lines, where both the chemical shield (anther cuticle) and the physical shield (petal trichome) were synchronously abolished or enhanced. Thus, it is proposed that *GhMYB106_D13* functions as a central developmental hub that directly coordinates the allocation of epidermal resources, constructing a multilayered physical and chemical architecture to sequester anthers from environmental stresses.

In summary, *GhMYB106_D13* functions as a master regulator of a ‘dual‐shield’ defense system. It constructs a microscopic ‘first shield’ by activating the VLCFA and alkane biosynthetic pathways to seal the anther cuticle against desiccation and UV radiation. Simultaneously, it coordinates a macroscopic ‘second shield’ by promoting the initiation of trichomes that physically enclose the floral bud. This cooption of a single transcription factor to manage both chemical and physical barriers represents a vital evolutionary adaptation for reproductive success.

## MATERIALS AND METHODS

### Plant materials and growth conditions

The interspecific introgression lines M247, EH191 were derived from the recurrent parent Emian22 (E22, an upland cotton cultivar) crossed with *G. barbadense* acc. 3‐79 (AD_1_), and *G. mustelinum* (AD_4_), respectively. The upland cotton accession Jin668 was used as the receptor for *Agrobacterium*‐mediated genetic transformation (Jin et al., 2006). Two F_2_ mapping populations derived from the crosses M247 × E22 and M247 × 3‐79 were utilized for genetic mapping of the D13 and A13 loci, respectively. All cotton materials were grown in the experimental field at Huazhong Agricultural University, Wuhan, Hubei Province, China, under standard field management.

### DNA and RNA extraction and quantitative reverse transcriptase‐polymerase (qRT‐PCR)

Genomic DNA was isolated from young cotton leaves using a CTAB method. For gene expression analysis, anthers were collected at different developmental stages as previously described (Wu et al., 2015). Total RNA was extracted using the RNAprep Pure Plant Kit (TIANGEN Biotech, Beijing, China) according to the manufacturer's instructions. First‐strand cDNA was synthesized from 1 μg of total RNA using M‐MLV Reverse Transcriptase (Promega, Madison, WI, USA). The relative expression levels were calculated using the 2^−ΔΔCt^ method, with *GhUBQ7* (*Ghir_A11G011460*) serving as the internal reference. All primers used in this study are listed in Table S1.

### Construction of vectors

For stable transformation in cotton, the full‐length genomic DNA sequence of *GhMYB106_D13* (excluding the promoter region) was amplified and cloned into the pGWB415 vector downstream of the CaMV 35S promoter using Gateway technology (Invitrogen, Carlsbad, CA, USA). *Ghmyb106_D13* mutant was created by CRISPR/Cas9 system using pRGEB32‐GhU6.9 (Wang, Zhang, et al., 2018) as the knock‐out vector with the target sequence TAGCTTATATTGAAGAACAT. The OE and knock‐out vectors were introduced into *G. hirsutum* acc. Jin668 by *Agrobacterium tumefaciens*‐mediated transformation. For subcellular localization assays, the full‐length CDS of *GhMYB106_D13* was inserted into the pGWB741 vector. For dual‐luciferase reporter assays, the promoter sequences of target genes were cloned into the pGreenII 0800‐LUC vector between the *Hin*dIII and *Nco*I sites to generate reporter constructs. The CDS of *GhMYB106_D13* was cloned into the pGreenII 62‐SK vector between the *Bam*HI and *Hin*dIII sites to serve as the effector.

### Subcellular localization and dual‐luciferase assays

The constructed vectors were transformed into *A. tumefaciens* strain GV3101. For transient expression, bacteria were infiltrated into the leaves of 3‐week‐old *Nicotiana benthamiana* plants. The infiltrated plants were cultivated at 25°C with a 16‐h light/8‐h dark photoperiod for 48–72 h. For subcellular localization, GFP fluorescence signals were observed using a confocal laser scanning microscope (Olympus FV1200, Tokya, Japan). Nuclei were visualized by staining with 4′,6‐diamidino‐2‐phenylindole (DAPI). For dual‐LUC assays, firefly luciferase (LUC) and renilla luciferase (REN) activities were measured using a cryogenically cooled CCD camera (Lumazome PyLoN 2048B, Trenton, NJ, USA).

### Scanning electron microscopy

Fresh anthers at Stage‐12 were collected from Jin668, *GhMYB106_D13* KO lines, and OE lines. Samples were carefully detached and immediately fixed in 2.5% (v/v) glutaraldehyde at 4°C overnight. The samples were then dehydrated through a graded ethanol series (30, 50, 70, 80, 90, 95, and 100%), critical point dried with liquid CO_2_, and sputter‐coated with gold and then proceeded to scanning electron microscope.

### Cuticular wax extraction and GC‐MS analysis

The surface areas of cotton anthers were calculated based on microscopic measurements. For wax extraction, approximately 1 g of fresh anthers was immersed in 10 ml of chloroform spiked with 10 μg of *n*‐tetracosane (C24) as an internal standard. Extraction was facilitated by ultrasonication for 30 sec. The extract was transferred, and the anthers were re‐extracted twice with fresh chloroform. The combined supernatants were filtered and dried under a gentle stream of nitrogen gas at 40°C. The dried residue was weighed to determine the total wax load.

For compositional analysis, the dried wax extract was derivatized by adding 25 μl of pyridine and 25 μl of *N*,*O*‐bis (trimethylsilyl) trifluoroacetamide (BSTFA) containing 1% trimethylchlorosilane (TMCS). The mixture was incubated at 70°C for 45 min. After evaporating the derivatization reagents under nitrogen gas, the samples were reconstituted in 1 ml of *n*‐hexane and filtered through a 0.22‐μm membrane. The samples were analyzed by gas chromatography‐mass spectrometry (GC‐MS).

### RNA sequencing (RNA‐seq) and data analysis

Total RNA was extracted from Stage‐8 and Stage‐12 anthers (Wu et al., 2015) of Jin668, *GhMYB106_D13* KO lines and OE lines with two to three biological replicates per genotype. Sequencing libraries were constructed and sequenced on an Illumina HiSeq 2500 platform. Clean reads were mapped to the *G. hirsutum* TM‐1 reference genome (Li et al., 2024; Zhang et al., 2015) using HISAT2 software (version 2.0). Gene read counts were calculated using FeatureCounts, and differential expression analysis was performed using the DESeq2 R package. DEGs were identified based on a threshold of |log_2_ (fold change)| >1 and a Benjamini–Hochberg adjusted *P*‐value <0.05.

### Water loss and water permeability assays

For water loss measurements, approximately 1 g of Stage‐12 anthers was collected from each genotype simultaneously. Samples were equilibrated at room temperature for 1 h to remove surface moisture and then placed in an incubator at 28°C. Weights were recorded at 0, 6, and 12 h, and water loss was calculated as the percentage of initial fresh weight. For cuticle permeability assays, Stage‐12 anthers were immersed in deionized water at 30°C for 48 h. Subsequently, the anthers were stained with 0.05% (w/v) toluidine blue for 5 min, rinsed gently with water to remove excess dye, and photographed immediately.

## AUTHOR CONTRIBUTIONS

ZL and XZ conceived and designed the project. BG conducted the genetic mapping and transformation. BG and MC conducted the phenotypic characterization and other experiments. BG and CF analyzed the sequencing data. BG drafted the manuscript.

## CONFLICT OF INTEREST

The authors declare no conflicts of interest.

## Supporting information


**Figure S1.** Sequence analysis of GhMYB106_D13 and its homologs.

**Figure S2.** Phenotype of plants including Jin668, knockout line, and overexpression line.

**Figure S3.** The GO and KEGG analyses in Jin668, over‐expression line, and knockout line.

**Figure S4.** Trichome phenotype of Jin668, knockout line, and overexpression line.

**Figure S5.** The GO and KEGG analyses in TM‐1 and *ob1*.

**Table S1.** Molecular markers and primers used in this study.
**Table S2.** Comparison of the expressed genes in the mapping region with those in the collinear regions of AD1‐A13 and AD2‐D13.
**Table S3.** Analysis of the MYB binding sites of promoter by PlantCARE.
**Table S4.** DEGs in the petal transcriptomes of TM‐1 and ob1.

## Data Availability

The RNA‐seq datasets generated during the current study are available in the Genome Sequence Archive (GSA) at the National Genomics Data Center under accession number CRA037765 (https://ngdc.cncb.ac.cn/gsa/search?searchTerm=CRA037765).
