# Supplementary material for: GhMYB106_D13 coordinates cuticle biosynthesis to build the dual shields for anther protection in upland cotton
Source: Plant J. 2026 Oct 7;128(1):e71142. doi: 10.1111/tpj.71142 (PMC13646479; doi:10.1111/tpj.71142)
Supplement: Supplementary file 1 [file TPJ-128-0-s001.pdf]

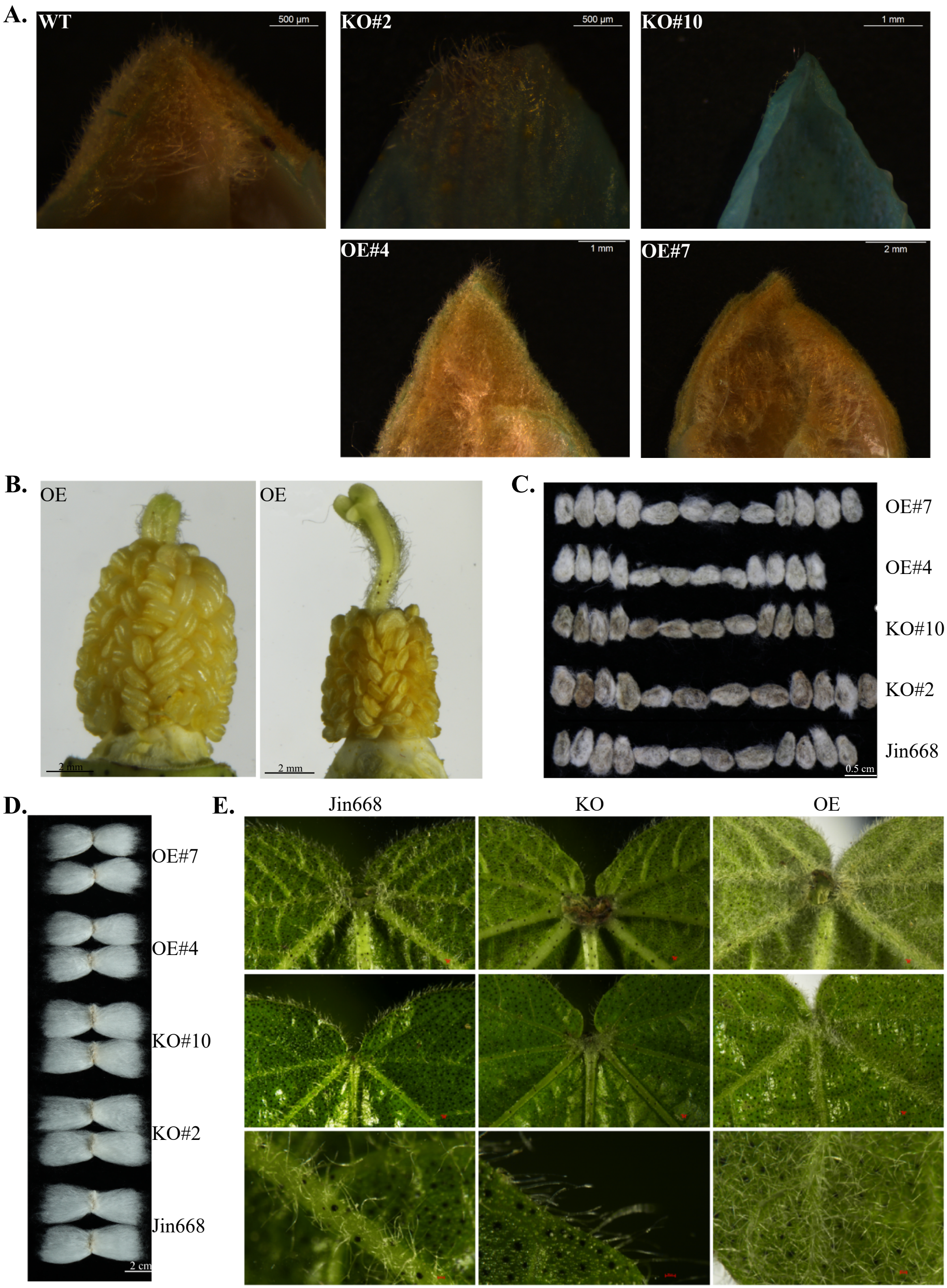

A.
WT
500 µm
KO#2
500 µm
KO#10
1 mm
OE#4
1 mm
OE#7
2 mm
B.
OE
2 mm
OE
2 mm
C.
OE#7
OE#4
KO#10
KO#2
Jin668
0.5 cm
D.
OE#7
OE#4
KO#10
KO#2
Jin668
2 cm
E.
Jin668
KO
OE
