# Supplementary material for: GhMYB106_D13 coordinates cuticle biosynthesis to build the dual shields for anther protection in upland cotton
Source: Plant J. 2026 Oct 7;128(1):e71142. doi: 10.1111/tpj.71142 (PMC13646479; doi:10.1111/tpj.71142)
Supplement: Supplementary file 2 [file TPJ-128-0-s002.pdf]

A.

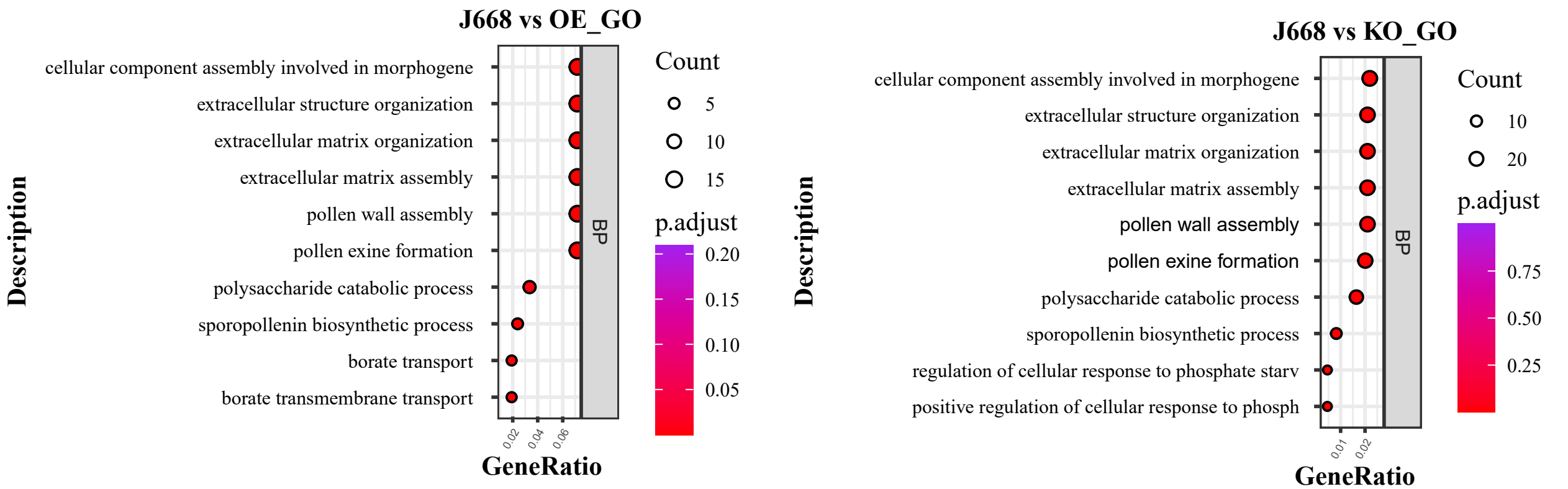
J668 vs OE_GO
cellular component assembly involved in morphogene
extracellular structure organization
extracellular matrix organization
extracellular matrix assembly
pollen wall assembly
pollen exine formation
polysaccharide catabolic process
sporopollenin biosynthetic process
borate transport
borate transmembrane transport
Description
BP
0.02
0.04
0.06
GeneRatio
Count
5
10
15
p.adjust
0.20
0.15
0.10
0.05
J668 vs KO_GO
cellular component assembly involved in morphogene
extracellular structure organization
extracellular matrix organization
extracellular matrix assembly
pollen wall assembly
pollen exine formation
polysaccharide catabolic process
sporopollenin biosynthetic process
regulation of cellular response to phosphate starv
positive regulation of cellular response to phosph
Description
BP
0.01
0.02
GeneRatio
Count
10
20
p.adjust
0.75
0.50
0.25

B.

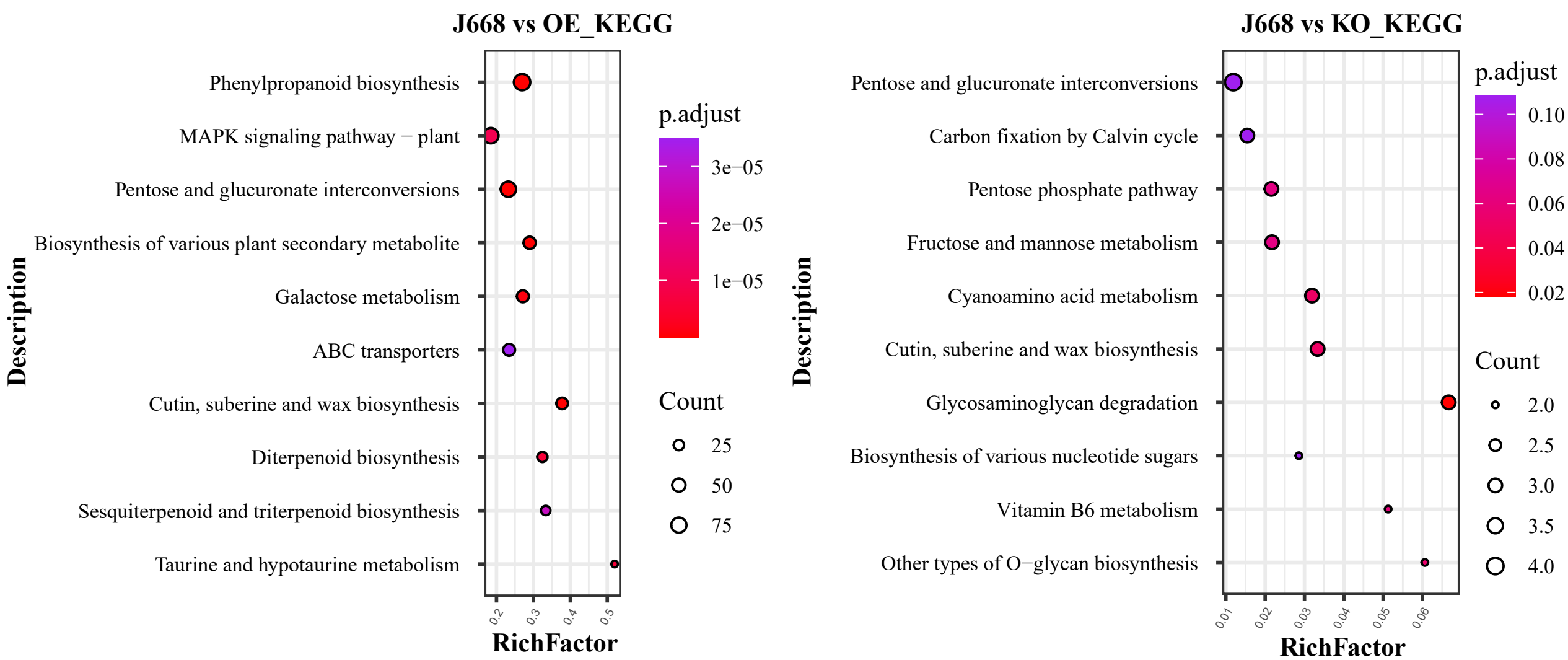
J668 vs OE_KEGG
Phenylpropanoid biosynthesis
MAPK signaling pathway − plant
Pentose and glucuronate interconversions
Biosynthesis of various plant secondary metabolite
Galactose metabolism
ABC transporters
Cutin, suberine and wax biosynthesis
Diterpenoid biosynthesis
Sesquiterpenoid and triterpenoid biosynthesis
Taurine and hypotaurine metabolism
Description
0.2
0.3
0.4
0.5
RichFactor
p.adjust
3e−05
2e−05
1e−05
Count
25
50
75
J668 vs KO_KEGG
Pentose and glucuronate interconversions
Carbon fixation by Calvin cycle
Pentose phosphate pathway
Fructose and mannose metabolism
Cyanoamino acid metabolism
Cutin, suberine and wax biosynthesis
Glycosaminoglycan degradation
Biosynthesis of various nucleotide sugars
Vitamin B6 metabolism
Other types of O−glycan biosynthesis
Description
0.01
0.02
0.03
0.04
0.05
0.06
RichFactor
p.adjust
0.10
0.08
0.06
0.04
0.02
Count
2.0
2.5
3.0
3.5
4.0
