# Supplementary material for: GhMYB106_D13 coordinates cuticle biosynthesis to build the dual shields for anther protection in upland cotton
Source: Plant J. 2026 Oct 7;128(1):e71142. doi: 10.1111/tpj.71142 (PMC13646479; doi:10.1111/tpj.71142)

A.

```
E22   AAATAATAACAAAAAATAAGGGCTTCAAAGAT
3-79  AAATAATAACAATAAATAAAGGCTTCAAAGAT
M247  AAATAATAACAATAAATAAAGGCTTCAAAGAT
      ************ ****** ************
```

B.

```
Gh_MYB106   MADCREKMGLKKGPWTPDEDQKLLAYIEEHGLGNWRTLPEKAGLQRCGKSCRLRWINYLR
Grai_MYB106 MADCREKMGLKKGPWTPDEDQKLLAYIEEHGLGNWRTFPEKAGLQRCGKSCRLRWINYLR
Gar_MYB106  MADCREKMGLKKGPWTPDEDQKLLAYFEEHGLGNWRTLPEKAGLQRCGKSCRLRWINYLR
            **************************:**********:**********************

Gh_MYB106   PDLKRGKFSLQEEQTIIQLHAFLGNRWSTIAAHLPNRTDNEIKNYWNTHVKKRFTKMGID
Grai_MYB106 PDLKRGKFSLQEEQTIIQLHAFLGNRWSTIAAHLPNRTDNEIKNYWNTHVKKRFTKMGID
Gar_MYB106  PDLKRGKFSLQEEQTIIQLHAFLGNRWSTIAAHLPNRTDNEIKNYWNTHIKKRFTKMGID
            *************************************************:**********

Gh_MYB106   PTTHKPKSNHVVSPTGRTTLNHMAQWESARLEAEARLVKDSKNLPSSSSRPSPYQKSCNK
Grai_MYB106 PTTHKPKSNHVVSPTGRTTLNHMAQWESARLEAEARLVKDSKNLPSSSSRPSPYQKSCNK
Gar_MYB106  PTTHKPKSNHVVNPTGRTTVNHMAQWESARLEAEARLVKDSKKLPSSSSRPSPYQKSCNK
            ************.******:**********************:*****************

Gh_MYB106   GSKSQCLDVVKAWQSVVAGMFATSTNNSNRIIFGPDQSSGNYELDSIIPIGGNVEDELMV
Grai_MYB106 GSKSQCLDVVKAWQSVVAGMFATSTNNSNRIIFGPDQSSGNYELDSIIPIGGNVEDELMV
Gar_MYB106  GSKSQCLDVVKAWQSVVAGMFATSTNNSNRIIFGPDQSSGNYELDSIIPLGGNVEDELMV
            *************************************************:**********

Gh_MYB106   GNDRSKCQVPELNERFDNYMSLHDTTHLWAAPIAENDVVEGFPDFLVHDFDYQIDNEESI
Grai_MYB106 GNDRSKCQVPELNERFDNYMSLHDTTHLWAAPIAENDVVEGFPDFLVHDFDYQIDNEESI
Gar_MYB106  GNDRSKCQVPELNERFDNYMSLHDTTHLWAAPIAENDVVESFPDFLVHDFDYQIDNEESI
            ****************************************.*******************

Gh_MYB106   TV
Grai_MYB106 TV
Gar_MYB106  T-
            *
```

C.

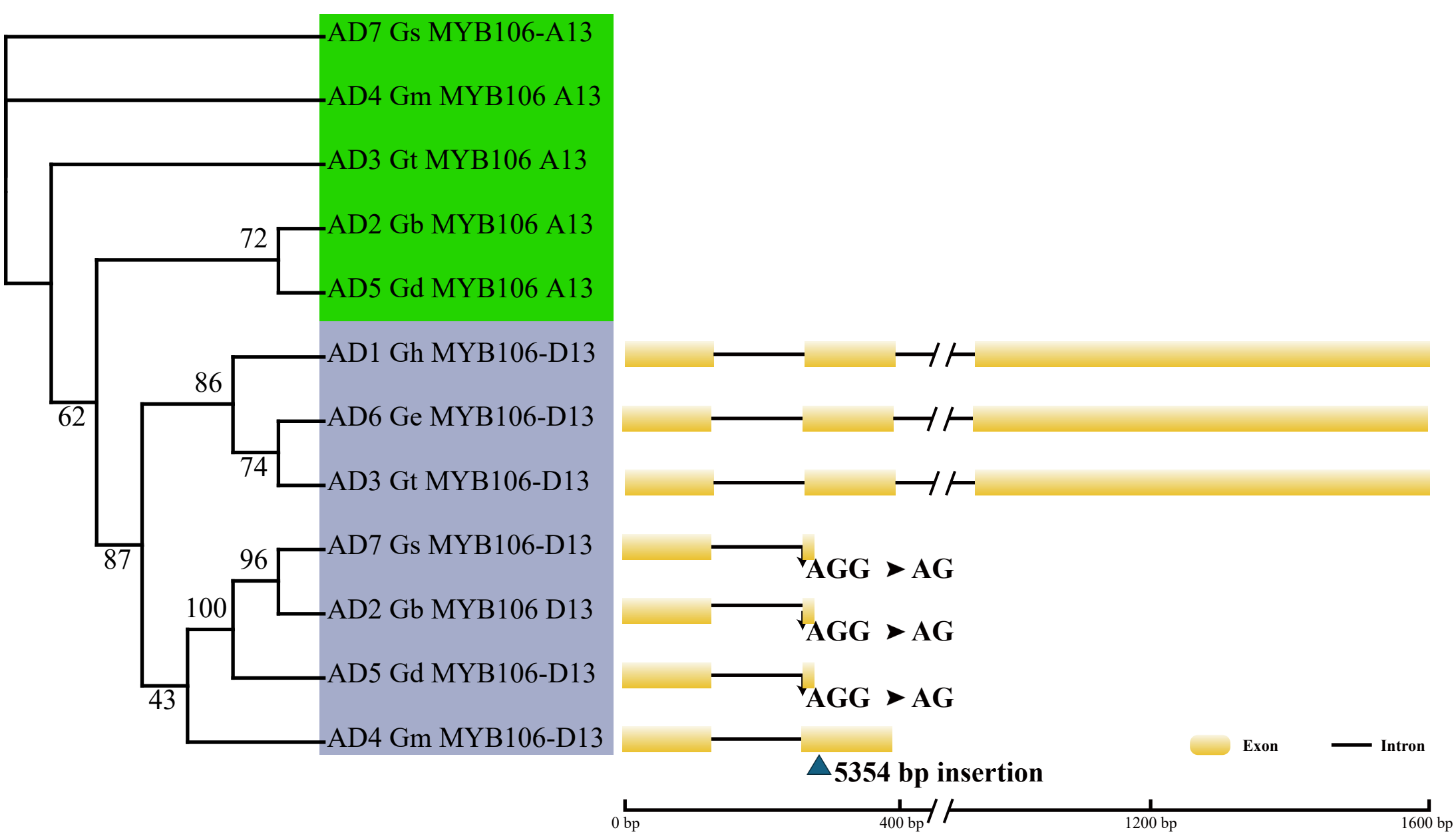

Supplement: Supplementary file 3 [file TPJ-128-0-s004.pdf]
