# Supplementary material for: GhMYB106_D13 coordinates cuticle biosynthesis to build the dual shields for anther protection in upland cotton
Source: Plant J. 2026 Oct 7;128(1):e71142. doi: 10.1111/tpj.71142 (PMC13646479; doi:10.1111/tpj.71142)
Supplement: Supplementary file 4 [file TPJ-128-0-s005.pdf]

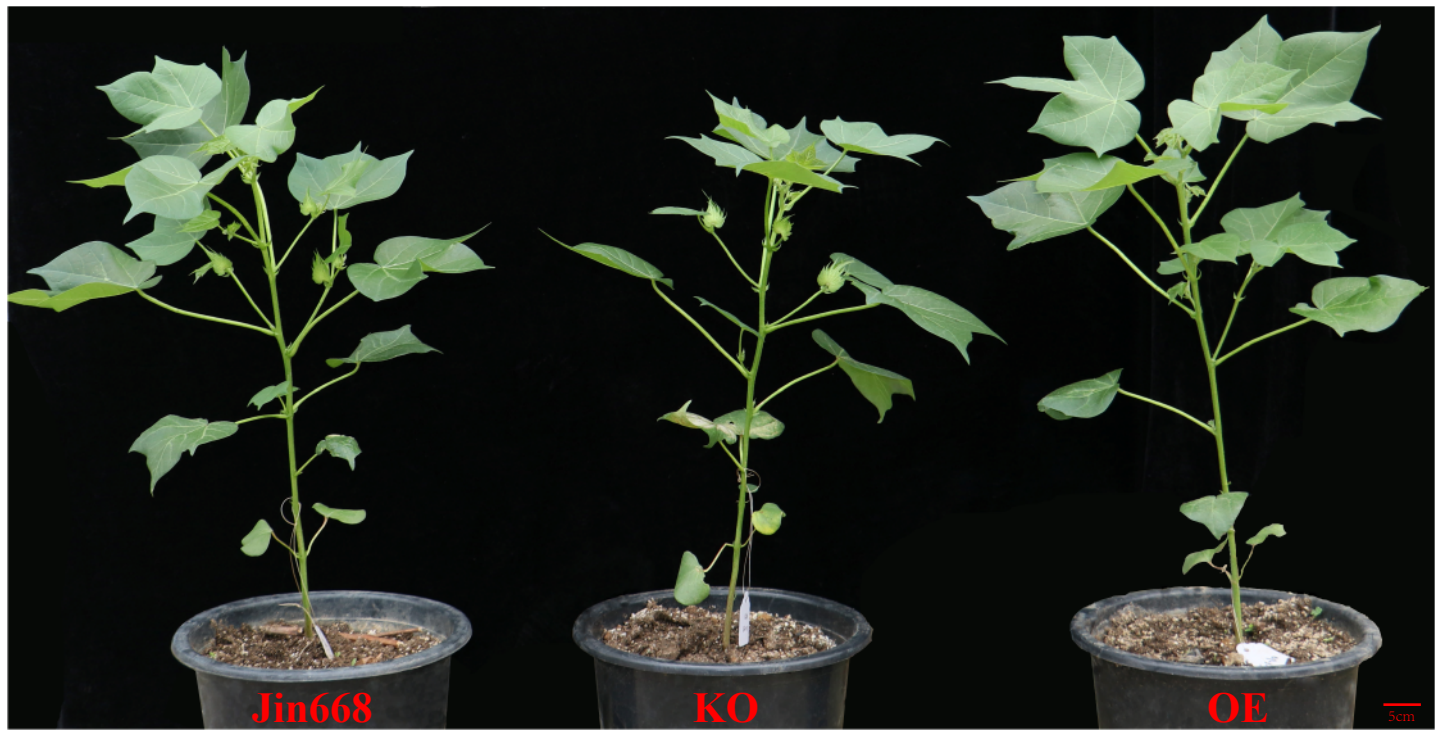
Jin668
KO
OE
5cm
