# Supplementary material for: GhMYB106_D13 coordinates cuticle biosynthesis to build the dual shields for anther protection in upland cotton
Source: Plant J. 2026 Oct 7;128(1):e71142. doi: 10.1111/tpj.71142 (PMC13646479; doi:10.1111/tpj.71142)
Supplement: Supplementary file 5 [file TPJ-128-0-s006.pdf]

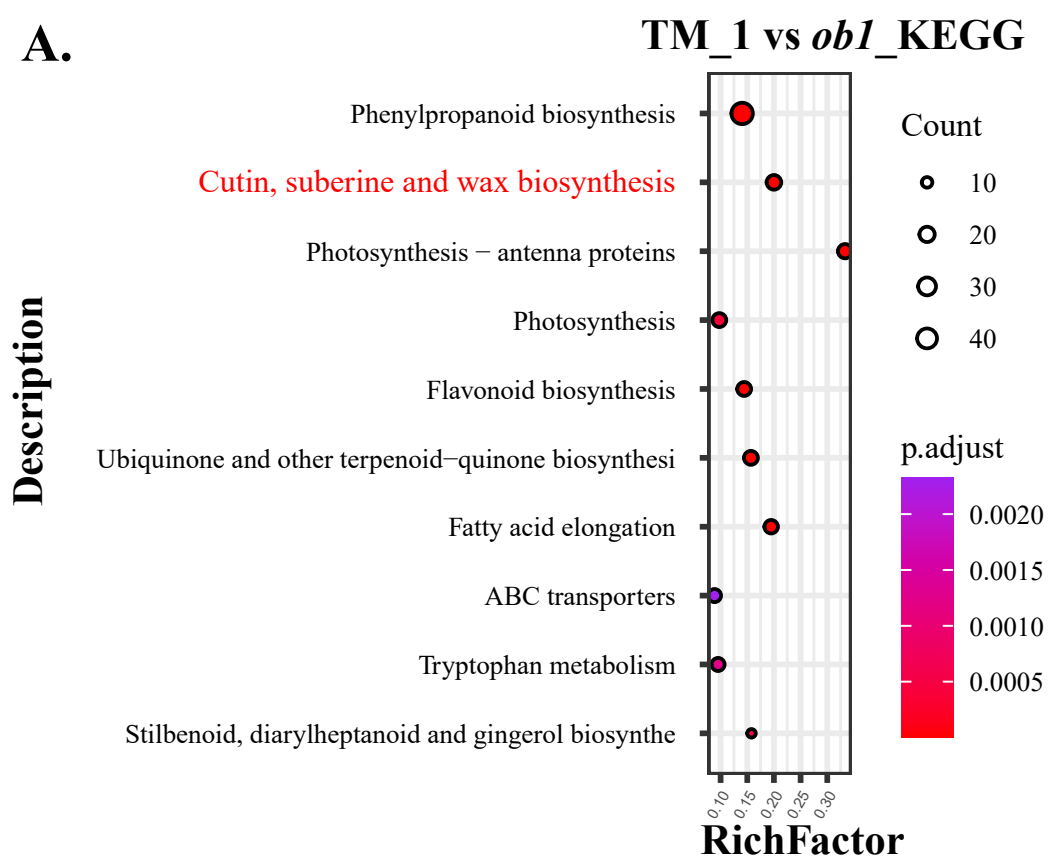
A.
TM_1 vs ob1_KEGG
Description
Phenylpropanoid biosynthesis
Cutin, suberine and wax biosynthesis
Photosynthesis − antenna proteins
Photosynthesis
Flavonoid biosynthesis
Ubiquinone and other terpenoid−quinone biosynthesi
Fatty acid elongation
ABC transporters
Tryptophan metabolism
Stilbenoid, diarylheptanoid and gingerol biosynthe
RichFactor
Count
10
20
30
40
p.adjust
0.0020
0.0015
0.0010
0.0005

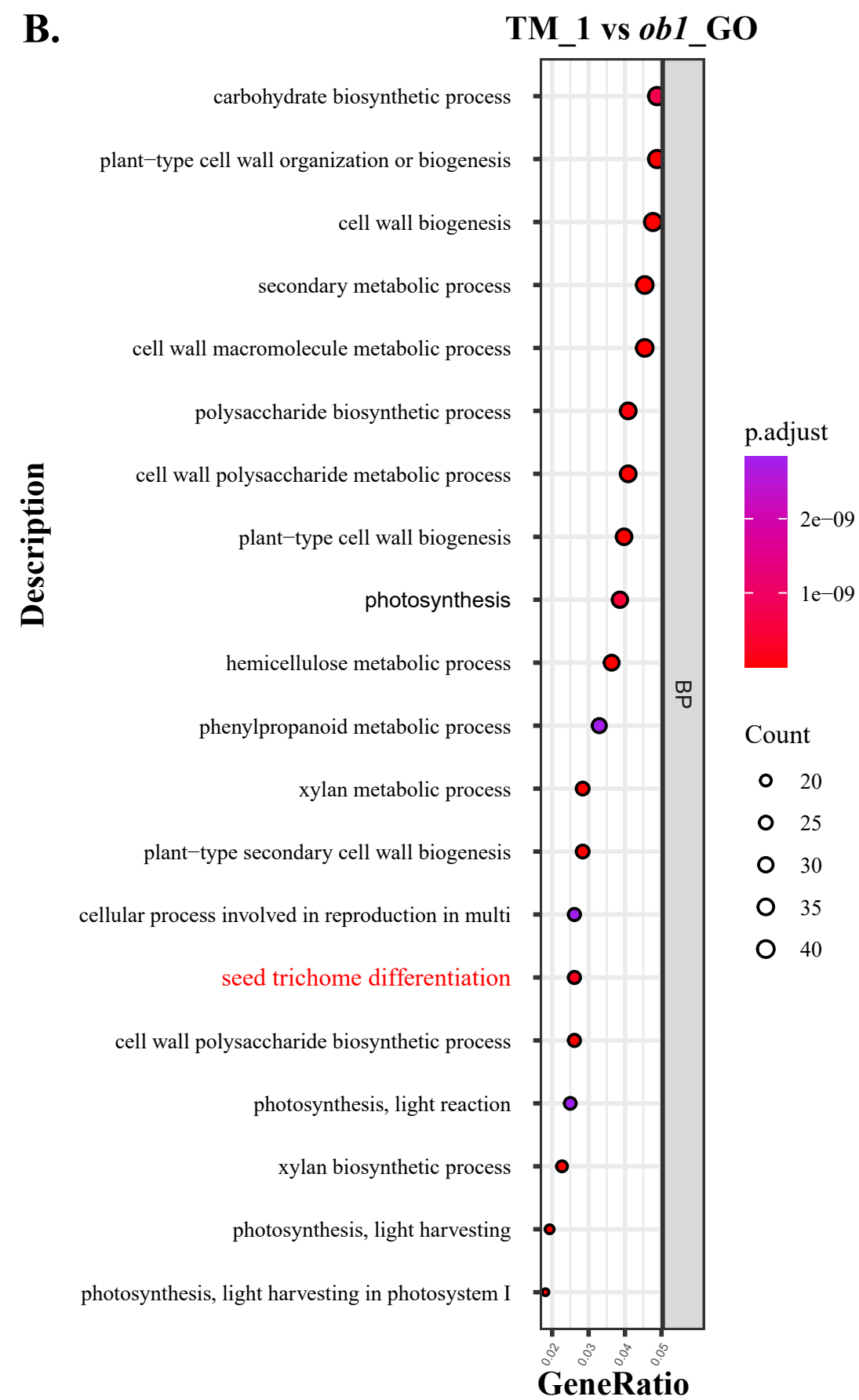
B.
TM_1 vs ob1_GO
Description
carbohydrate biosynthetic process
plant−type cell wall organization or biogenesis
cell wall biogenesis
secondary metabolic process
cell wall macromolecule metabolic process
polysaccharide biosynthetic process
cell wall polysaccharide metabolic process
plant−type cell wall biogenesis
photosynthesis
hemicellulose metabolic process
phenylpropanoid metabolic process
xylan metabolic process
plant−type secondary cell wall biogenesis
cellular process involved in reproduction in multi
seed trichome differentiation
cell wall polysaccharide biosynthetic process
photosynthesis, light reaction
xylan biosynthetic process
photosynthesis, light harvesting
photosynthesis, light harvesting in photosystem I
BP
GeneRatio
0.02
0.03
0.04
0.05
p.adjust
2e−09
1e−09
Count
20
25
30
35
40
